# Double Omental Hernia: A Case Report and Literature Review

**DOI:** 10.7759/cureus.94775

**Published:** 2025-10-17

**Authors:** Ana M Fernandes, Bárbara Gaspar, João Curado, Susana Costa, Eduardo Coutinho

**Affiliations:** 1 General Surgery, Unidade Local de Saúde Póvoa de Varzim/Vila do Conde, Póvoa de Varzim, PRT; 2 General Surgery, Hospital das Forças Armadas, Porto, PRT; 3 General Surgery, Centro Hospitalar do Tâmega e Sousa, Guilhufe, PRT

**Keywords:** acute abdomen, double omental hernia, internal hernia, omental defect, small bowel obstruction

## Abstract

Double omental hernia is an exceptionally rare type of internal herniation, in which small bowel loops pass through two distinct defects in the omentum, usually involving both the gastrocolic and gastrohepatic ligaments. Its clinical presentation is often nonspecific, frequently mimicking other causes of acute abdomen and delaying diagnosis. We report a case of a 71-year-old man with a previous near-total colectomy for familial adenomatous polyposis, admitted with acute abdominal pain and radiologic signs suggestive of pneumoperitoneum. Emergency laparotomy revealed a double omental internal hernia, with small bowel herniation through the gastrocolic and hepatogastric ligaments, resulting in obstruction and bowel perforation. Surgical treatment consisted of hernia reduction, segmental enterectomy, and closure of the omental defects. The patient had an uneventful recovery. This case highlights the importance of recognizing a double omental hernia as a potential cause of acute abdomen, especially in patients with previous abdominal surgery.

## Introduction

Internal hernias are a rare cause of intestinal obstruction, accounting for approximately 0.2-0.9% of all abdominal hernias and between 0.5% and 5.8% of all cases of intestinal occlusion [1,2]. They involve the protrusion of the intestine through peritoneal or mesenteric defects within the abdominal cavity, without the formation of an external hernial sac. Among the different subtypes, omental hernias are particularly uncommon, and double omental hernias (involving simultaneous defects in both the gastrocolic and gastrohepatic ligaments) are exceptionally rare, with only a few cases described in the literature [2,3].

The double omental hernia may arise congenitally or develop secondary to inflammation, trauma, or previous surgery that compromises the integrity of the omental attachments [2,4,5]. Because the omentum forms part of both the greater and lesser peritoneal sacs, a defect in each structure can allow communication between these cavities, predisposing to internal herniation of small bowel loops.

This report describes a case of double omental hernia presenting as an acute abdomen in a patient with a previous colectomy. The clinical presentation, intraoperative findings, and possible pathophysiological mechanisms are discussed in light of current literature.

## Case presentation

Patient information and medical history

A 71-year-old male had a history of laparoscopic near-total colectomy for familial adenomatous polyposis performed five years earlier, which was complicated by a postoperative abscess requiring reoperation via laparotomy. He also had a history of radiotherapy for prostate cancer four years earlier and hypertension.

Clinical presentation

The patient presented to the emergency department with diffuse abdominal pain of sudden onset five days earlier, initially intermittent but progressively worsening, and associated with diarrhea and abdominal distension. There was no vomiting or fever. On examination, the abdomen was distended with tenderness and moderate guarding, more pronounced in the right abdominal quadrants. Laboratory tests showed mild anemia and elevated inflammatory markers. An abdominopelvic CT scan revealed pneumoperitoneum and a right paracolic fluid collection, suggesting a visceral perforation, although its origin was not clear.

Surgical findings

**Figure 1:**
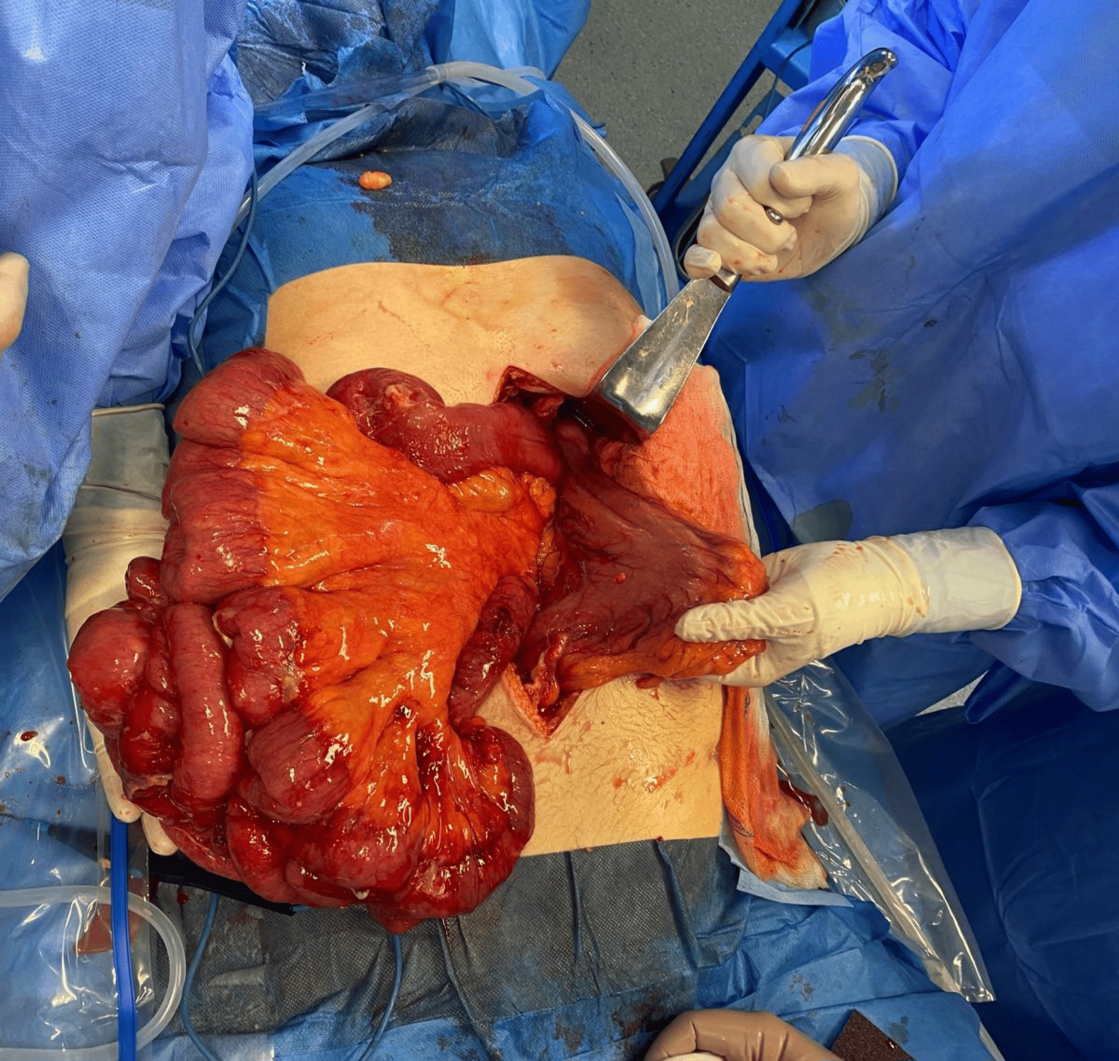
Intraoperative image showing small bowel loops herniating through both omental defects.

Emergency laparoscopic exploration was initiated but converted to an open midline laparotomy due to limited visualization and poor exposure. Intraoperatively, as shown in Figure 1, a double omental hernia was identified. The first defect was located in the gastrocolic ligament, through which distal ileal loops had herniated into the lesser sac. The second defect, in the hepatogastric (lesser omentum) ligament, allowed further passage of these loops into the supramesocolic compartment, effectively creating a closed-loop obstruction. The entrapped bowel segment demonstrated congestion and focal perforation, consistent with ischemic compromise.

The herniated bowel loops were reduced, and the nonviable segment was resected with primary anastomosis performed. Both omental defects were closed to prevent recurrence. The abdomen was thoroughly irrigated, and bilateral drains were placed.

Postoperative course

The postoperative course was favorable. The patient gradually resumed oral intake and was discharged on postoperative day 7 with full recovery.

## Discussion

Internal hernias are rare but potentially life-threatening causes of small bowel obstruction, often diagnosed during surgical exploration [3]. Among them, omental hernias, whether through congenital or acquired defects, constitute a minute fraction. The double omental hernia, described in this case, is even more uncommon and characterized by herniation through sequential defects in the greater and lesser omenta [3].

Pathogenesis

The omentum functions as a protective, vascularized peritoneal fold separating the greater and lesser sacs. When a defect develops in the gastrocolic ligament (anterior boundary of the lesser sac) and another in the hepatogastric ligament (superior boundary), small bowel loops can pass from the greater sac into the lesser sac and re-enter the peritoneal cavity, creating a “double-gated” herniation [4,6]. In this patient, the presence of postoperative adhesions and prior intra-abdominal infection likely contributed to secondary weakening or tearing of the omental layers, facilitating the formation of acquired defects. Such iatrogenic or inflammatory alterations are well-documented predisposing factors for internal hernias in patients with prior abdominal surgery [1-2,5].

Diagnosis

Preoperative diagnosis of omental hernias remains difficult due to nonspecific clinical findings. CT is the most reliable imaging modality, capable of revealing dilated bowel loops, transition points, or clusters of small bowel posterior to the stomach, features that suggest a transomental or lesser sac hernia [1,2]. However, in most reported cases, definitive diagnosis is achieved intraoperatively, as occurred in this case.

Management

Prompt surgical intervention is critical to prevent strangulation and ischemic necrosis [4]. The principles of management include reduction of the herniated loops, resection of nonviable bowel, and closure of all omental defects [5]. Laparoscopic management is feasible in selected patients, but conversion to open laparotomy may be necessary when visualization is compromised or when perforation is present.

Prognosis

Early recognition and timely surgical treatment result in excellent outcomes. Delayed diagnosis, however, can lead to bowel necrosis, perforation, and peritonitis, substantially increasing morbidity and mortality [2]. In this case, prompt intervention and definitive repair led to full recovery.

## Conclusions

Double omental hernia is an exceptionally rare but important differential diagnosis of acute abdomen, particularly in patients with prior abdominal surgery. Awareness of this entity, combined with prompt surgical exploration, is critical to prevent bowel ischemia and perforation. Recognition of possible predisposing factors, such as postoperative adhesions or inflammatory weakening of omental attachments, may help anticipate this rare complication. Early intervention ensures favorable postoperative recovery, as demonstrated in this case.
